# Housing and Food Insecurity, Health Literacy, and Maladaptive Coping Behaviors

**DOI:** 10.3928/24748307-20221019-01

**Published:** 2022-10

**Authors:** Patrece L. Joseph, Janelle Applewhite, Sasha A. Fleary

## Abstract

**Background:**

Stressors related to social determinants of health (SDH), such as housing and food insecurity, are implicated in chronic disease risk. Maladaptive strategies for coping with these stressors may exacerbate risk.

**Objective:**

Given the limited research on SDH-related stressors and maladaptive coping, this study examined the relationship between two SDH-related stressors (housing and food insecurity) and maladaptive coping behaviors (i.e., emotional eating and stress-related cigarette and alcohol use) in adults. Further, because health literacy (HL), another SDH, may be a protective factor, this study explored whether HL moderated these relationships.

**Methods:**

Data were collected from adults (*N* = 500, Mean age = 49.01 years, standard deviation = 16.36; 40% White) in the United States. A series of hierarchical logistic regressions predicting maladaptive coping behaviors from demographics, SDH-related stressors (i.e., housing or food insecurity), and HL variables were estimated.

**Key Results:**

Housing insecurity was associated with increased odds of emotional eating (odds ratio [OR] = 1.48, *p* < .001), stress-related cigarette use (*OR* = 1.34, *p* = .001), and stress-related alcohol use (*OR* = 1.32, *p* = .001). Food insecurity was associated with increased odds of emotional eating (*OR* = 1.49, *p* = .012), stress-related cigarette (*OR* = 1.68, *p* = .002), and stress-related alcohol use (*OR* = 1.49, *p* = .013). Higher functional HL scores were associated with decreased odds of emotional eating after accounting for housing (*OR* = 0.79, *p* = .017) and food insecurity (*OR* = 0.76, *p* = .004). Communicative and critical HL moderated the relationship between food insecurity and emotional eating.

**Conclusions:**

Examining HL in relation to SDH-related stressors and maladaptive coping behaviors is complex. HL may be less protective for maladaptive coping behaviors that are likely addictive. Because HL domains may require cooperation between individuals and systems related to stressors, multi-systemic interventions are necessary to reduce maladaptive coping behaviors. [***HLRP: Health Literacy Research and Practice*. 2022;6(4):e280–e289.**]

**Plain language summary:**

Stress related to inadequate resources for housing and food may be related to adults' poor coping behaviors (e.g., emotional eating and stress-related cigarette and alcohol use). Adults who experienced housing and food insecurity were more likely to report emotional eating and using cigarettes and alcohol when stressed. Adults with higher functional health literacy were less likely to report emotional eating.

The social determinants of health (SDH) are the structural and social conditions that affect one's health (Wilkinson & Marmot, 2003). The features of individuals' physical and social environments (e.g., access to secure housing and healthy foods) are well documented SDH linked to poor health outcomes. For example, food insecurity and inadequate access to healthy, affordable foods are linked to poor diet (Hanson & Connor, 2014; Leung et al., 2014), obesity (Pan et al., 2012), and cardiovascular risk factors (Seligman et al., 2010). Whereas housing insecurity, lack of access to stable, adequate, safe, and affordable housing (Cox et al., 2019), is associated with poor mental and physical health (Linton et al., 2021; Stahre et al., 2015). The stress associated with experiences of food and housing insecurity may exacerbate poor health outcomes via maladaptive coping behaviors (MCB) (i.e., emotional eating and stress-related substance use) (Marmot, 2005; Stahre et al., 2015). MCB may help individuals manage immediate stress, however, long-term engagement in MCB may increase risks for chronic health conditions (Nishimura et al., 2020; Spinosa et al., 2019) and further exacerbate stress. While efforts to address the structural conditions that result in housing and food security are vital, identifying modifiable, protective factors that may be leveraged in interventions to help individuals cope with SDH-related stressors is also critical.

Health literacy (HL), a modifiable skillset, may promote adaptive strategies for coping with stress. HL is the ability to obtain, understand, and utilize health information to engage in everyday decision-making about health care, disease prevention, and health promotion (Sørensen et al., 2012). Low HL is associated with high perceived stress among adults (Hoover et al., 2015; Michou et al., 2021). HL skills may also contribute to individuals' capacity to access and use resources to cope with SDH-related stressors. For example, functional HL, which involves reading and numerical skills, may be used to gather and apply information about coping with stress to one's daily life (Nutbeam, 2000). Communicative or interactive HL skills (i.e., social skills involved in health-related interactions) may be leveraged for social support or to identify resources to address stressors. Critical HL, skills for taking personal and social action on health, may help individuals advocate for resources for themselves and their communities (Nutbeam, 2000). Several researchers conceptualize HL skills as protective (Carroll et al., 2015; Herman & Jackson, 2010; Logan et al., 2015; Nutbeam, 2000). Among individuals that experience SDH-related stressors, those with high HL may be better equipped to cope with stressors in a healthy manner. To date, no studies have explored HL as a protective factor in the relationship between SDH-related stressors and MCB. If a protective factor, HL may be considered in short-term efforts to reduce MCB among individuals experiencing SDH-related stressors.

## Current Study

This study examined (1) the relationship between two SDH-related stressors, housing and food insecurity, and three types of MCB (emotional eating and stress-related cigarette and alcohol use); and (2) whether HL skills moderated the relationships between SDH-related stressors and MCB. We hypothesized that SDH-related stressors would be positively associated with MCB, and that this association would vary by participants' HL. Specifically, for individuals with higher (vs. lower) HL, the relationship between MCB and SDH-related stressors would be more negative and weaker.

## Methods

### Procedures

This study was approved by the Tufts University Social, Behavioral and Educational Research Institutional Review Board. Data were collected from March 2018 to October 2018 (*N* = 500). As contracted by the corresponding author, Qualtrics staff recruited participants from third-party research panels using stratified random sampling. Stratification was based on the distribution of demographic characteristics of the United States, with an oversampling for racial and ethnic minority groups. Qualtrics emailed survey links to participants who met the researchers' eligibility criteria (age: ≥18 years, resided in the U.S.). The survey link routed participants to the Qualtrics platform where they completed informed consent before completing the mental health attitudes and beliefs and HL survey. Participants completed the survey in about 30 minutes and responded to multiple attention-check questions dispersed throughout the survey. After survey completion, participants were routed to a debriefing form with mental health resources. Participants received incentives established by Qualtrics (e.g., gift cards, redeemable points). Data were also collected via social media (*n* = 101) but were excluded as the sample included non-U.S. respondents.

## Measures

### Demographic Covariates

Participants reported their age, gender, race, ethnicity, and education level. Options for gender were male, female, trans-gender, male-to-female transgender, female-to-male transgender, gender non-conforming, and other. Participants selected their race from the following options: Black/African American; Asian; Native American or Alaskan Native; Native Hawaiian or Other Pacific Islander; and White. They indicated whether they were of Hispanic, Latino/a/e, or Spanish origin. Participants selected their highest level of education completed. Options were less than high school, high school, some college, Associate's degree, Bachelor's degree, and Graduate degree.

### Food Insecurity

Participants responded to: Which of these statements best describes the food situation in your household in the past 12 months? This item was from the National Survey of Children's Health (Ghandour et al., 2018). Response options were, we could always afford to eat good nutritious meals, we could always afford enough to eat but not always the kinds of foods we should eat, sometimes we could not afford enough to eat, and often we could not afford enough to eat. Response options were coded 0 to 3, respectively.

### Housing Insecurity

Participants responded to: How often in the past 12 months would you say you were worried or stressed about having enough money to pay for your rent or mortgage? This item was from the Family Life, Activity, Sun, Health, and Eating (FLASHE) survey (Nebeling et al., 2017). Response options were *never*, *almost never*, *sometimes*, *fairly often*, and *very often*. Response options were coded to 0 to 4, respectively.

### Emotional Eating

Two items from the FLASHE study were used to assess emotional eating (Nebeling et al., 2017). Participants were asked how often they started or continued to eat when not hungry because they felt (1) sad or depressed and (2) anxious or nervous. Response options were *never*, *rarely*, *sometimes*, *often*, and *always*. Responses were coded into 0 (*never* or *rarely* for both items) and 1 (*sometimes*, *often*, or *always* for at least one item).

### Stress-Related Cigarette Use

Participants indicated whether they smoked at least 100 cigarettes in their lifetime and if they smoked cigarettes to help cope with stress. The former question was from the Behavioral Risk Factor Surveillance System (Centers for Disease Control and Prevention, 2014) and the latter question was developed for this study. Response options were *yes*, *no*, and *don't know*/*not sure*. This variable was dichotomized into no stress-related cigarette use (never smoked/no to latter question) and stress-related cigarette use (yes to latter question). Participants who indicated *don't know*/*not sure* (*n* = 7) were excluded from analyses.

### Stress-Related Alcohol Use

Four items from the Drinking Motives Questionnaire-Revised (Cooper, 1994) were used to assess participants' alcohol use. Participants indicated whether they ever drank alcohol in their lives. If yes, they were asked how often they had a drink in the last 12 months because it helps them when they feel depressed or nervous, to cheer up when they're in a bad mood, or to forget about their problems. Response options were *never*, *sometimes*, and *almost always*. Responses were dichotomized into no stress-related alcohol use (never drank alcohol or never to all three latter questions) and stress-related alcohol use (*sometimes*/*almost* *always* to at least 1 of the 3 latter questions).

### Health Literacy

The 13-item All Aspects of Health Literacy Scale (Chinn & McCarthy, 2013) assessed functional, communicative, and critical HL, and empowerment. Participants responded to three functional (e.g., When you need help, can you easily get ahold of someone to assist you?) and communicative HL questions (e.g., When you talk to a doctor or nurse, do you ask the questions you need to ask?); four critical HL questions (e.g., Are you someone who likes to find out lots of different information about your health?) and three empowerment questions (e.g., What do you think matters most for everyone's health?). Response options were *sometimes*, *often*, or *always* for the functional, communicative, and critical HL questions and varied for the empowerment questions. Variables were re-coded and scores were summed, with higher scores indicating higher HL. Score ranges were as follows: functional and communicative HL (3–9), critical HL (4–12), and empowerment (3–6). Cronbach's alpha reliability for the measure was 0.74.

### Data Analysis

Data analyses were completed in SPSS Version 27. Hierarchical logistic regression analyses were estimated to predict adults' MCB. Demographic variables were first entered into the model, then the SDH-related stressor (i.e., housing or food insecurity), and finally all of the HL variables. Next, models were estimated for each HL domain to assess each as a moderator between SDH-related stressors and MCB. Crossover interactions are significant when main effects are non-significant (Baron & Kenny, 1986); therefore, all HL interactions were assessed. Significant interaction terms were plotted for interpretation.

## Results

The sample included 500 adults whose average age was 49.01 years (standard deviation = 16.36; see **Table 1** for demographic characteristics). The largest racial group was White (about 40%). Approximately 10.4% of adults reported being worried or stressed about having enough money to pay their rent or mortgage *very often* and 8.6% of adults reported *sometimes* or *often* not being able to afford enough to eat. Housing and food insecurity were positively correlated (*r* = 0.49, *p* < .001). Approximately 39% of adults engaged in emotional eating and about 32% and about 28% in stress-related cigarette and alcohol use, respectively. The average score for the HL domains were as follows: functional = 7.98 (*SD* = 1.24), communicative = 8.22 (*SD* = 1.30), empowerment = 4.48 (*SD* = 0.81), and critical = 9.33 (*SD* = 2.00). Housing insecurity was inversely correlated with functional (r = −.25, *p* < .001) and communicative (r = −.17, *p* < .001) HL, and empowerment (r = −.12, *p* = .011). Food insecurity was inversely correlated with functional (r = −.16, *p* < .001), communicative (r = −.24, *p* < .001), and critical (r = −.12, *p* = .009) HL.

Results of the hierarchical logistic regressions with housing insecurity are presented in **Table 2**. Older adults were less likely to engage in emotional eating [*OR* = 0.97, 95% *CI* = 0.96, 0.98] and more likely to engage in stress-related cigarette use [*OR* = 1.02, 95% *CI* = 1.01, 1.04]. Compared to White adults, Asian adults were less likely to engage in stress-related cigarette use [*OR* = 0.34, 95% *CI* = 0.16, 0.72]. After controlling for demographics, housing insecurity was associated with increased odds of emotional eating [*OR* = 1.48, 95% *CI* = 1.25, 1.75], stress-related cigarette use [*OR* = 1.34, 95% *CI* = 1.12, 1.59], and stress-related alcohol use [*OR* = 1.32, 95% *CI* = 1.12, 1.57]. After controlling for demographics and housing insecurity, higher functional HL was associated with decreased odds of emotional eating [*OR* = 0.79, 95% *CI* = 0.65, 0.96].

**Table 1:**
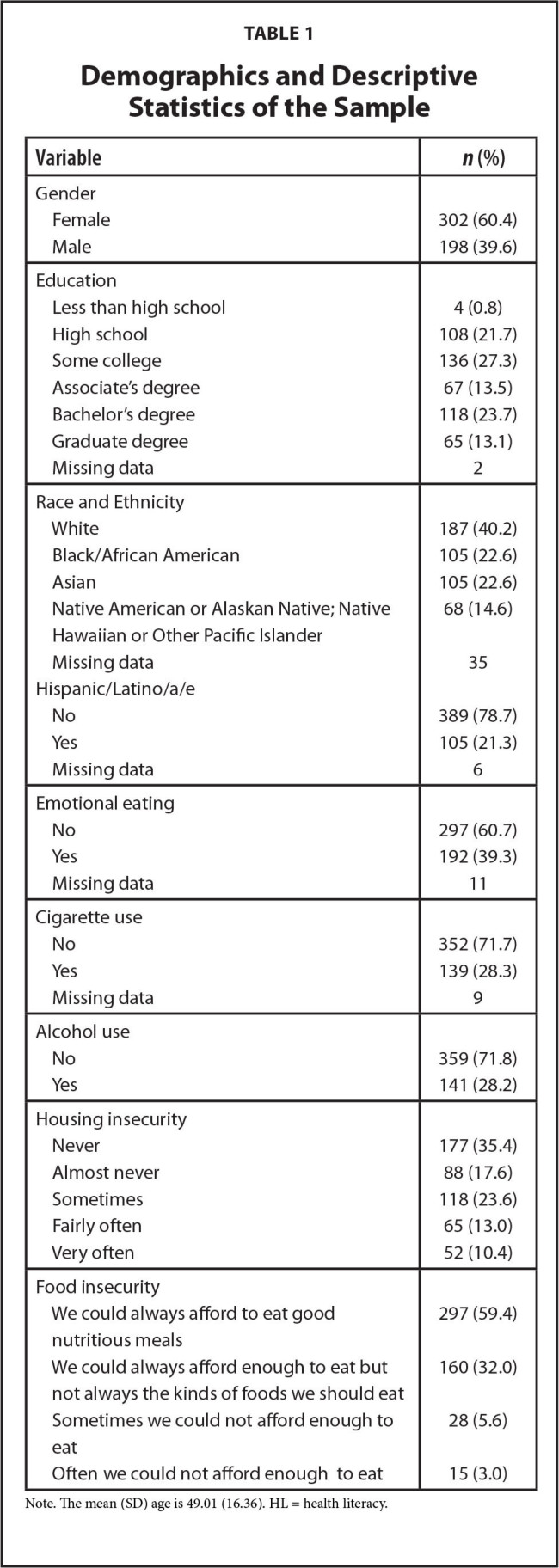
Demographics and Descriptive Statistics of the Sample

**Variable**	***n* (%)**

Gender	
Female	302 (60.4)
Male	198 (39.6)

Education	
Less than high school	4 (0.8)
High school	108 (21.7)
Some college	136 (27.3)
Associate's degree	67 (13.5)
Bachelor's degree	118 (23.7)
Graduate degree	65 (13.1)
Missing data	2

Race and Ethnicity	
White	187 (40.2)
Black/African American	105 (22.6)
Asian	105 (22.6)
Native American or Alaskan Native; Native	68 (14.6)
Hawaiian or Other Pacific Islander Missing data	35
Hispanic/Latino/a/e	
No	389 (78.7)
Yes	105 (21.3)
Missing data	6

Emotional eating	
No	297 (60.7)
Yes	192 (39.3)
Missing data	11

Cigarette use	
No	352 (71.7)
Yes	139 (28.3)
Missing data	9

Alcohol use	
No	359 (71.8)
Yes	141 (28.2)

Housing insecurity	
Never	177 (35.4)
Almost never	88 (17.6)
Sometimes	118 (23.6)
Fairly often	65 (13.0)
Very often	52 (10.4)

Food insecurity	
We could always afford to eat good nutritious meals	297 (59.4)
We could always afford enough to eat but not always the kinds of foods we should eat	160 (32.0)
Sometimes we could not afford enough to eat	28 (5.6)
Often we could not afford enough to eat	15 (3.0)

Note. The mean (SD) age is 49.01 (16.36). HL = health literacy.

**Table 2:**
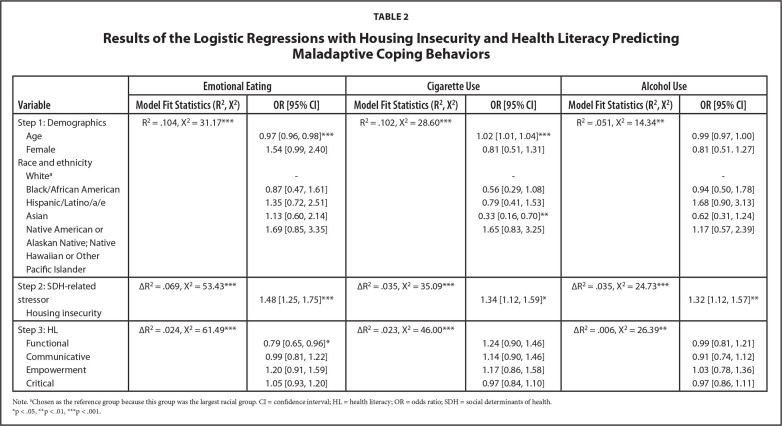
Results of the Logistic Regressions with Housing Insecurity and Health Literacy Predicting Maladaptive Coping Behaviors

**Variable**	**Emotional Eating**		**Cigarette Use**		**Alcohol Use**	

	**Model Fit Statistics (R^2^, X^2^)**	**OR [95% CI]**	**Model Fit Statistics (R^2^, X^2^)**	**OR [95% CI]**	**Model Fit Statistics (R^2^, X^2^)**	**OR [95% CI]**

Step 1: Demographics	R^2^= .104, X^2^= 31.17^***^	0.97 [0.96, 0.98]^***^	R^2^= .102, X^2^= 28.60^***^	1.02 [1.01, 1.04]^***^	R^2^= .051, X^2^= 14.34^**^	0.99 [0.97, 1.00]
Age						
Female		1.54 [0.99, 2.40]		0.81 [0.51, 1.31]		0.81 [0.51. 1.27]
Race and ethnicity						
White^a^		-		-		-
Black/African American		0.87 [0.47, 1.61]		0.56 [0.29, 1.08]		0.94 [0.50, 1.78]
Hispanic/Latino/a/e		1.35 [0.72, 2.51]		0.79 [0.41, 1.53]		1.68 [0.90, 3.13]
Asian		1.13 [0.60, 2.14]		0.33 [0.16, 0.70]^**^		0.62 [0.31, 1.24]
Native American or Alaskan Native; Native Hawaiian or Other Pacific Islander		1.69 [0.85, 3.35]		1.65 [0.83, 3.25]		1.17 [0.57, 2.39]

Step 2: SDH-related stressor	∆R^2^= .069, X^2^= 53.43^***^	1.48 [1.25, 1.75]^***^	∆R^2^= .035, X^2^= 35.09^***^	1.34 [1.12, 1.59]^*^	∆R^2^= .035, X^2^= 24.73^***^	1.32 [1.12, 1.57]^**^
Housing insecurity						

Step 3: HL	∆R^2^= .024, X^2^= 61.49^***^	0.79 [0.65, 0.96]^*^	∆R^2^= .023, X^2^= 46.00^***^	1.24 [0.90, 1.46]	∆R^2^= .006, X^2^= 26.39^**^	0.99 [0.81, 1.21]
Functional						
Communicative		0.99 [0.81, 1.22]		1.14 [0.90, 1.46]		0.91 [0.74, 1.12]
Empowerment		1.20 [0.91, 1.59]		1.17 [0.86, 1.58]		1.03 [0.78, 1.36]
Critical		1.05 [0.93, 1.20]		0.97 [0.84, 1.10]		0.97 [0.86, 1.11]

Note.

a Chosen as the reference group because this group was the largest racial group. CI = confidence interval; HL = health literacy; OR = odds ratio; SDH = social determinants of health.

* p < .05,

** p < .01,

*** p < .001.

Results of the hierarchical logistic regressions with food insecurity are presented in **Table 3**. Demographics were identical to those presented in **Table 2**. After controlling for demographics, food insecurity was associated with increased odds of emotional eating [*OR* =1.49, 95% *CI* = 1.09, 2.03], and stress-related cigarette [*OR* = 1.68, 95% *CI* = 1.21, 2.34] and stress-related alcohol use [*OR* = 1.49, 95% *CI* = 1.09, 2.03]. After controlling for demographics and food insecurity, higher functional HL was associated with decreased odds of emotional eating [*OR* = 0.76, 95% *CI* = 0.62, 0.91].

**Table 3:**
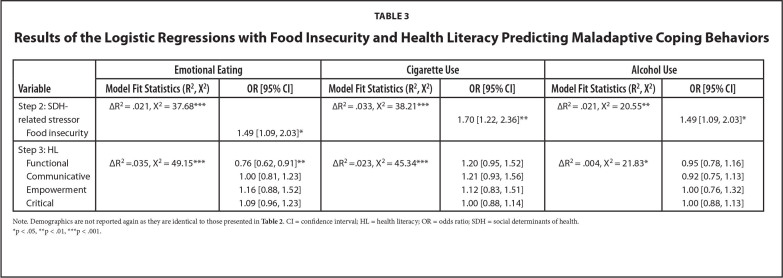
Results of the Logistic Regressions with Food Insecurity and Health Literacy Predicting Maladaptive Coping Behaviors

**Variable**	**Emotional Eating**		**Cigarette Use**		**Alcohol Use**	

	**Model Fit Statistics (R^2^, X^2^)**	**OR [95% CI]**	**Model Fit Statistics (R^2^, X^2^)**	**OR [95% CI]**	**Model Fit Statistics (R^2^, X^2^)**	**OR [95% CI]**

Step 2: SDH-related stressor	ΔR^2 ^= .021, X^2 ^= 37.68^***^	1.49 [1.09, 2.03]^*^	ΔR^2 ^= .033, X^2 ^= 38.21^***^	1.70 [1.22, 2.36]^**^	ΔR^2 ^= .021, X^2 ^= 20.55^**^	1.49 [1.09, 2.03]^*^
Food insecurity						

Step 3: HL						
Functional	ΔR^2 ^=.035, X^2 ^= 49.15^***^	0.76 [0.62, 0.91]^**^	ΔR^2 ^=.023, X^2 ^= 45.34^***^	1.20 [0.95, 1.52]	ΔR^2 ^= .004, X^2 ^= 21.83^*^	0.95 [0.78, 1.16]
Communicative		1.00 [0.81, 1.23]		1.21 [0.93, 1.56]		0.92 [0.75, 1.13]
Empowerment		1.16 [0.88, 1.52]		1.12 [0.83, 1.51]		1.00 [0.76, 1.32]
Critical		1.09 [0.96, 1.23]		1.00 [0.88, 1.14]		1.00 [0.88, 1.13]

Note. Demographics are not reported again as they are identical to those presented in Table 2. CI = confidence interval; HL = health literacy; OR = odds ratio; SDH = social determinants of health.

* p < .05,

** p < .01,

*** p < .001.

### HL as Moderators

Results of the hierarchical logistic regressions with interactions for each HL domain and housing and food insecurity are presented in **Tables 4** and **5**, respectively. Only communicative [*OR* = 1.22, 95% *CI* = 1.04, 1.43] and critical HL [*OR* = 1.17, 95% *CI* = 1.02, 1.34] moderated the relationships between food insecurity and emotional eating. All other interactions were non-significant.

**Table 4:**
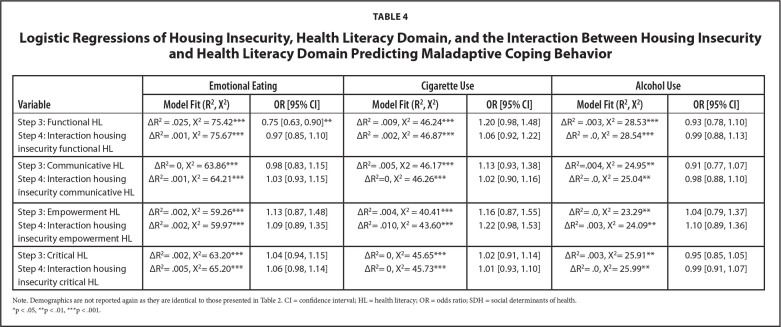
Logistic Regressions of Housing Insecurity, Health Literacy Domain, and the Interaction Between Housing Insecurity and Health Literacy Domain Predicting Maladaptive Coping Behavior

**Variable**	**Emotional Eating**		**Cigarette Use**		**Alcohol Use**	

	**Model Fit (R^2^, X^2^)**	**OR [95% CI]**	**Model Fit (R^2^, X^2^)**	**OR [95% CI]**	**Model Fit (R^2^, X^2^)**	**OR [95% CI]**

Step 3: Functional HL	ΔR^2 ^= .025, X^2 ^= 75.42^***^	0.75 [0.63, 0.90]^**^	ΔR^2 ^= .009, X^2 ^= 46.24^***^	1.20 [0.98, 1.48]	ΔR^2 ^= .003, X^2 ^= 28.53^***^	0.93 [0.78, 1.10]
Step 4: Interaction housing insecurity functional HL	ΔR^2^= .001, X^2 ^= 75.67^***^	0.97 [0.85, 1.10]	ΔR^2 ^= .002, X^2 ^= 46.87^***^	1.06 [0.92, 1.22]	ΔR^2 ^= .0, X^2 ^= 28.54^***^	0.99 [0.88, 1.13]

Step 3: Communicative HL	ΔR^2^= 0, X^2 ^= 63.86^***^	0.98 [0.83, 1.15]	ΔR^2^= .005, X2 = 46.17^***^	1.13 [0.93, 1.38]	ΔR^2^=.004, X^2 ^= 24.95^**^	0.91 [0.77, 1.07]
Step 4: Interaction housing insecurity communicative HL	ΔR^2^= .001, X^2 ^= 64.21^***^	1.03 [0.93, 1.15]	ΔR^2^=0, X^2 ^= 46.26^***^	1.02 [0.90, 1.16]	ΔR^2^= .0, X^2 ^= 25.04^**^	0.98 [0.88, 1.10]

Step 3: Empowerment HL	ΔR^2^= .002, X^2 ^= 59.26^***^	1.13 [0.87, 1.48]	ΔR^2^= .004, X^2 ^= 40.41^***^	1.16 [0.87, 1.55]	ΔR^2^= .0, X^2 ^= 23.29^**^	1.04 [0.79, 1.37]
Step 4: Interaction housing insecurity empowerment HL	ΔR^2^= .002, X^2 ^= 59.97^***^	1.09 [0.89, 1.35]	ΔR^2^= .010, X^2 ^= 43.60^***^	1.22 [0.98, 1.53]	ΔR^2^= .003, X^2 ^= 24.09^**^	1.10 [0.89, 1.36]

Step 3: Critical HL	ΔR^2^= .002, X^2^= 63.20^***^	1.04 [0.94, 1.15]	ΔR^2^= 0, X^2^= 45.65^***^	1.02 [0.91, 1.14]	ΔR^2^= .003, X^2^= 25.91^**^	0.95 [0.85, 1.05]
Step 4: Interaction housing insecurity critical HL	ΔR^2^= .005, X^2^= 65.20^***^	1.06 [0.98, 1.14]	ΔR^2^= 0, X^2^= 45.73^***^	1.01 [0.93, 1.10]	ΔR^2^= .0, X^2^= 25.99^**^	0.99 [0.91, 1.07

Note. Demographics are not reported again as they are identical to those presented in Table 2. CI = confidence interval; HL = health literacy; OR = odds ratio; SDH = social determinants of health.

* p < .05,

** p < .01,

*** p < .001.

**Table 5:**
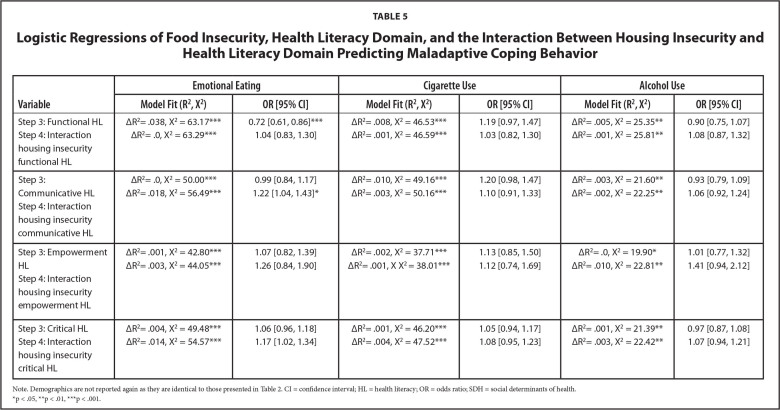
Logistic Regressions of Food Insecurity, Health Literacy Domain, and the Interaction Between Housing Insecurity and Health Literacy Domain Predicting Maladaptive Coping Behavior

**Variable**	**Emotional Eating**		**Cigarette Use**		**Alcohol Use**	

	**Model Fit (R^2^, X^2^)**	**OR [95% CI]**	**Model Fit (R^2^, X^2^)**	**OR [95% CI]**	**Model Fit (R^2^, X^2^)**	**OR [95% CI]**

Step 3: Functional HL	ΔR^2^= .038, X^2 ^= 63.17^***^	0.72 [0.61, 0.86]^***^	ΔR^2^= .008, X^2 ^= 46.53^***^	1.19 [0.97, 1.47]	ΔR^2^= .005, X^2 ^= 25.35^**^	0.90 [0.75, 1.07]
Step 4: Interaction housing insecurity functional HL	ΔR^2^= .0, X^2 ^= 63.29^***^	1.04 [0.83, 1.30]	ΔR^2^= .001, X^2 ^= 46.59^***^	1.03 [0.82, 1.30]	ΔR^2^= .001, X^2 ^= 25.81^**^	1.08 [0.87, 1.32]

Step 3: Communicative HL	ΔR^2^= .0, X^2 ^= 50.00^***^	0.99 [0.84, 1.17]	ΔR^2^= .010, X^2 ^= 49.16^***^	1.20 [0.98, 1.47]	ΔR^2^= .003, X^2 ^= 21.60^**^	0.93 [0.79, 1.09]
Step 4: Interaction housing insecurity communicative HL	ΔR^2^= .018, X^2 ^= 56.49^***^	1.22 [1.04, 1.43]^*^	ΔR^2^= .003, X^2 ^= 50.16^***^	1.10 [0.91, 1.33]	ΔR^2^= .002, X^2 ^= 22.25^**^	1.06 [0.92, 1.24]

Step 3: Empowerment HL	ΔR^2^= .001, X^2 ^= 42.80^***^	1.07 [0.82, 1.39]	ΔR^2^= .002, X^2 ^= 37.71^***^	1.13 [0.85, 1.50]	ΔR^2^= .0, X^2 ^= 19.90^*^	1.01 [0.77, 1.32]
Step 4: Interaction housing insecurity empowerment HL	ΔR^2^= .003, X^2 ^= 44.05^***^	1.26 [0.84, 1.90]	ΔR^2^= .001, X X^2 ^= 38.01^***^	1.12 [0.74, 1.69]	ΔR^2^= .010, X^2 ^= 22.81^**^	1.41 [0.94, 2.12]

Step 3: Critical HL	ΔR^2^= .004, X^2 ^= 49.48^***^	1.06 [0.96, 1.18]	ΔR^2^= .001, X^2 ^= 46.20^***^	1.05 [0.94, 1.17]	ΔR^2^= .001, X^2 ^= 21.39^**^	0.97 [0.87, 1.08]
Step 4: Interaction housing insecurity critical HL	ΔR^2^= .014, X^2 ^= 54.57^***^	1.17 [1.02, 1.34]	ΔR^2^= .004, X^2 ^= 47.52^***^	1.08 [0.95, 1.23]	ΔR^2^= .003, X^2 ^= 22.42^**^	1.07 [0.94, 1.21]

Note. Demographics are not reported again as they are identical to those presented in Table 2. CI = confidence interval; HL = health literacy; OR = odds ratio; SDH = social determinants of health.

* p < .05,

** p < .01,

*** p < .001.

**Figure 1.**
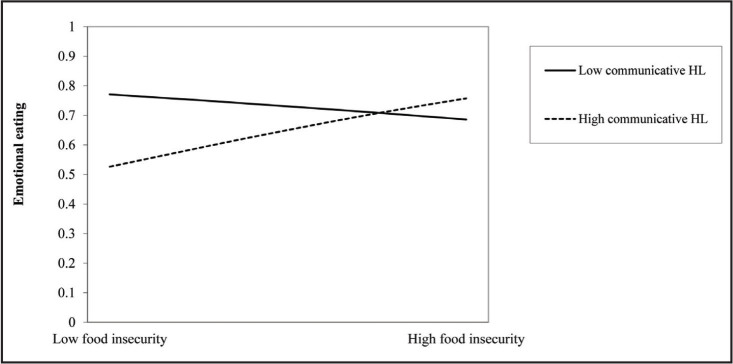
Communicative health literacy (HL) as a moderator in the relationship between food insecurity and emotional eating. Note. Plotted interaction of communicative health literacy and food insecurity predicting odds of emotional eating. Sociodemographic variables (i.e., age, gender, and race) were included in the model. At low food insecurity, adults with high communicative HL were less likely to engage in emotional eating than adults with low communicative HL. As food insecurity increased, adults with high (vs. low) communicative HL were more likely to engage in emotional eating.

At low food insecurity, having higher (vs. lower) communicative HL was associated with lower emotional eating. As food insecurity increased, having higher (vs. lower) communicative HL was associated with higher emotional eating (**Figure 1**). The gap between high and low communicative HL and the likelihood of emotional eating was larger at low (compared to high) food insecurity.

**Figure 2.**
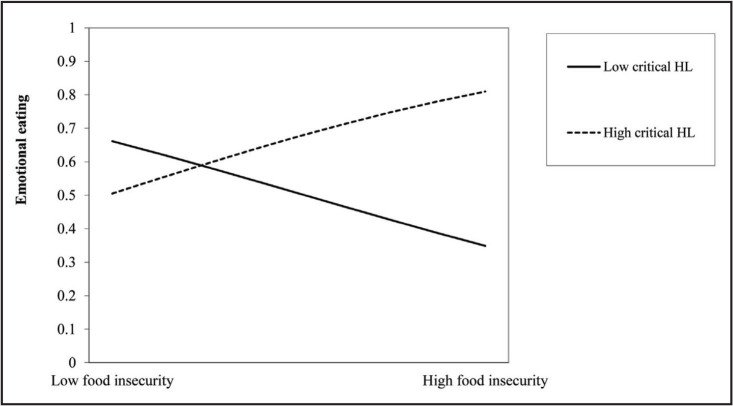
Critical health literacy (HL) as a moderator in the relationship between food insecurity and emotional eating. Note. Plotted interaction of critical health literacy and food insecurity predicting odds of emotional eating. Sociodemographic variables (i.e., age, gender, and race) were included in the model. At low food insecurity, adults with low critical HL were more likely to engage in emotional eating than adults with high critical HL. As food insecurity increased, adults with high (vs. low) critical HL were more likely to engage in emotional eating.

At low food insecurity, having higher (compared to lower) critical HL was associated with lower emotional eating. As food insecurity increased, having higher (vs. lower) critical HL was associated with higher emotional eating (**Figure 2**). The gap between high and low critical HL and the likelihood of emotional eating was smaller at low (compared to high) food insecurity.

## Discussion

The purpose of this study was to examine the relationship between SDH-related stressors and MCB and whether HL moderated this relationship. Our hypotheses were partially supported; housing and food insecurity were significantly associated with MCB. Functional HL was associated with emotional eating. Communicative and critical HL moderated the relationship between food insecurity and emotional eating. Our findings suggest that the relationship between SDH-related stressors, HL, and MCB varies and likely depends on the SDH-related stressor and MCB.

In our sample, housing and food insecurity were associated with increased odds of emotional eating and stress-related cigarette and alcohol use, providing further support for the prolonged and pervasive effects of these stressors on long-term health (Spinosa et al., 2019; Stahre et al., 2015). These findings highlight the importance of contextualizing individuals' health behaviors within their experience of the SDH, a sentiment echoed in the HL literature (Crondahl & Eklund Karlsson, 2016; Kendir & Breton, 2020). These results also support addressing SDH-related stressors to impact individual health. For example, hospital-community partnerships have improved housing to reduce children's exposure to inadequate housing in their communities (Kelleher et al., 2018). Screening families for SDH-related stressors at pediatric well visits has also been effective in helping families access resources to address their social needs (Garg et al., 2015; Gottlieb et al., 2016). The influence of these programs on MCB should be directly assessed. Further, combining these systemic interventions with individual-level stress management interventions may reduce harm caused by exposure to SDH-related stressors.

Relatedly, these systemic interventions may work best if communicative and critical HL and empowerment are high. These domains of HL go beyond the individual and may only serve the individual if there is cooperation from the systems that are required to intervene on the stressors. For example, having high communicative HL may not impact housing insecurity-related stressors if there are no systems to communicate with to effectively cope with housing insecurity. Instead, maladaptive coping might be high because the individual is aware of the limitations of their personal agency. This may explain why only functional HL was significant in the main effects models.

Functional HL is implicated in health risk knowledge and accessing health-related services and resources (Berkman et al., 2011; Sørensen et al., 2012), which might explain its negative relationship with emotional eating after controlling for demographics and stressors. Similarly, because HL is implicated in accessing resources and engagement in preventive health (Berkman et al., 2011; Estacio et al., 2019), the relationship between SDH-related stressors and MCB would be weaker or negative when HL was higher versus lower. We found the opposite results for communicative and critical HL regarding the relationship between food insecurity and emotional eating. Although individuals may have skills for accessing, understanding, and applying health information, there are other factors that reduce HL as a protective factor (e.g., availability of resources, psychological factors).

Higher HL skills may facilitate using resources and information to engage in health behavior but due to systemic barriers, these skills may not reduce SDH-related stressors. For instance, the application process for state and federal benefits programs are complicated (Kaye et al., 2013) and the resources provided may be insufficient for meeting housing and food needs (Bingulac et al., 2017; Crandall et al., 2021). Policy-level changes and alterations to administrative processes are needed to ensure individuals have resources for housing and food. Building collective action among individuals may be useful for addressing systemic injustices that result in SDH-related stressors as these strategies have increased communities' access to healthy, fresh foods (Freudenberg et al., 2011; White, 2011). Because critical HL skills include political and social action around health (Nutbeam, 2000; Sykes et al., 2013), enhancing individuals' critical HL skills may be an intermediary step in building collective action to address SDH-related stressors.

The lack of association between HL and stress-related alcohol and cigarette use is noteworthy. Stress-related substance use is qualitatively different from emotional eating due to the stronger likelihood of addiction to these substances. Stress may be a trigger for the behaviors, and because of the predisposition to addiction, the focus may be less on taking action to reduce the stressor and more on fulfilling the craving brought on by the stressor. Hence, HL may be a weak protective factor against this classification of MCB.

## Study Limitations

The present study has several limitations. The HL measure asks individuals to report on past behaviors and is subject to social desirability and recall biases. Future studies should use test-based measures of HL to ensure objective assessment of HL skills. Single-item measures of housing and food insecurity limit the validity of these constructs. However, these single-item screeners are used in national studies (e.g., FLASHE). Relatedly, the food insecurity item assessed participants' current experience whereas the housing insecurity item assessed participants' perceived stress. Future studies should include more robust measures that assess both current experience and perceived stress related to food and housing insecurity. Because this study was cross-sectional, the directionality of the relationships between the variables cannot be determined; thus, longitudinal studies are needed. Lastly, study participants were part of an opt-in online panel; therefore, findings may not be generalizable beyond individuals with high internet use and who willingly regularly participate in research studies.

## Conclusions

This study explored the relationship between SDH-related stressors, HL, and MCB. Both housing and food insecurity were negatively associated with MCB. Functional HL was associated with emotional eating, while communicative and critical HL moderated the relationship between food insecurity and emotional eating in an unexpected manner. The results highlight the complexity of studying HL in relation to SDH-stressors and MCB. Specifically, HL may be less protective for MCB that are likely addictive. Additionally, HL domains that require cooperation between the individual and systems related to the stressors may not be good intervention targets to protect individuals from maladaptive coping, particularly when the interventions are individualistic. Multisystemic interventions are necessary to reduce MCB.
